# Identification of an easy to use 3D culture model to investigate invasion and anticancer drug response in chondrosarcomas

**DOI:** 10.1186/s12885-017-3478-z

**Published:** 2017-07-18

**Authors:** Eva Lhuissier, Céline Bazille, Juliette Aury-Landas, Nicolas Girard, Julien Pontin, Martine Boittin, Karim Boumediene, Catherine Baugé

**Affiliations:** 1 Normandie Université, UNICAEN, EA7451 BioConnecT, 14032 Caen, France; 2 Service d’Anatomie Pathologique, CHU de Caen, Caen, France

**Keywords:** Alginate, Chondrosarcomas, Cancer, 3 dimensional culture, Antitumoral drug

## Abstract

**Background:**

Cytotoxic efficacy of anticancer drugs has been widely studied with monolayer-cultured cancer cells. However, the efficacy of drugs under two-dimensional (2D) culture condition usually differs from that of three-dimensional (3D) one. In the present study, an in vitro tumor tissue model was constructed using alginate hydrogel, and in vitro cytotoxic efficacy of two anticancer drugs (cisplatin and DZNep) was investigated in chondrosarcomas, and compared to in vivo response.

**Methods:**

Three cell lines derived from human chondrosarcomas, CH2879, JJ012 and SW1353, were embedded in alginate hydrogel. Proliferation and survival were assayed by ATP measurement using Cell Titer-Glo luminescent cell viability assay kit, and by counting viable cells in beads. Collagen and COMP expression was determined by RT-PCR. Invasion/migration was estimated by counting cells leaving alginate beads and adhering to culture dish. Then, chondrosarcoma response to cisplatin and DZNep was compared between cells cultured in monolayer or embedded in alginate, and using chondrosarcoma xenografts in nude mice.

**Results:**

Chondrosarcomas survived at least for 8 weeks, after embedment in alginate. However, only CH2879 cells could proliferate. Also, this cell line is more invasive than SW1353 and JJ012, which was coherent with the grade of their respective primary tumors. Furthermore, the expression of type II collagen was higher in chondrosarcomas cultured in 3D than in 2D. Interestingly, this 3D culture system allows to validate the absence of response of chondrosarcomas to cisplatin, and to predict the efficiency of DZNep to reduce chondrosarcoma growth in vivo.

**Conclusions:**

This study validates alginate beads as a relevant 3D model to study cancer biology and tumor responses to biological treatments.

## Background

Chondrosarcoma (CHS) is a malignant tumor characterized by the presence of a cartilaginous extracellular matrix. It represents the second most common primary bone tumor, and generally arises in adults aged between 30 to 70 years. The treatment consists on resection of the tumor, because of its resistance to conventional radiotherapy and chemotherapy [1, 2]. This resistance is linked to endogenous and external factors, such as mutations of genes involved in DNA repair and apoptosis [3, 4], or tumoral microenvironment [5]. In particular, the cartilaginous extracellular matrix (ECM) around chondrosarcomas reduces drug diffusion, restraining their delivery to the tumor cells [6].

Most studies aiming to investigate chondrosarcoma biology and response to anticancer drugs are done using chondrosarcoma cell lines cultured in monolayer. Whereas this method is useful to understand some processes and mechanisms, it often fails to mimic the natural tumor microenvironment, which is an important parameter in tumor signaling and drug response [7, 8]. In two-dimensional (2D) culture systems, cells are forced to adopt a planar morphology [9], which may alter cell proliferation, migration, invasion, apoptosis, as well as matrix production. As a result, cells generally display a dramatic reduction of malignant phenotype when compared to the tumor [10]*,* and do not response to drugs as in in vivo conditions. In addition, in the case of chondrosarcomas, cells fail to produce their characteristic abundant hyaline extracellular matrix. Therefore, the traditional 2D cell cultures cannot ideally recapitulate in vivo physiological conditions [6, 11]*.*

At contrary, three-dimensional (3D) cell culture systems are more likely to mimic natural tumor microenvironment in vitro [12]. Cells can migrate and have cell-matrix interactions and cell–cell contacts in all directions, allowing cell responses that more closely mimic events occurring in-vivo during cancer formation and progression [12]. Thereby, tumoral cells that grow in a 3D environment, tend to develop shapes and phenotypes observed in vivo [13, 14], display higher aggressiveness, overexpress pro-angiogenic growth factors and acquire drug resistance [15, 16]. That is why 3D culture models are becoming essential tools in cancer research, notably for testing the efficacy of anticancer drugs.

Several 3D models are developed in oncology field, such as spheroids or matrix-embedded tumor cells [17, 18]. Spheroids which constitute the simplest in vitro 3D model, are known to permit endogenous ECM deposition, cell-cell matrix interactions, and to mimic physiological barriers to drug delivery in vivo. Another 3D culture model consists to embed cells in natural or synthetic substrates. Biomaterials such as matrigel, alginate or collagen I are biologically active scaffolds that provide exogenous biological signals regulating cell growth and response to drugs. Synthetic and inert matrices are also able to sustain cultures in close proximity, and enable accumulation of newly secreted and defined ECM proteins by the embedded cells. All these models offer the possibility to simultaneously incorporate different cell types, such as fibroblasts, endothelial cells, adipocytes or immune cells. These co-culture systems, as tumor slices or explant cultures, permit to mimic tumor heterogeneity. Indeed, in addition to tumor cells, these cancer-associated cell types produce the non-cellular fraction of the tumor microenvironment, composed by the extracellular matrix, growth factors, cytokines, chemokines and exosomes, and interact with tumoral cells to impact biological features such as proliferation, migration as well as cellular response to drugs. These co-culture systems combining stromal and tumoral cells seem to be the best methods to model heterotypic cell-cell interactions. However, the implementation of standardized co-cultures that include different cell types remains challenging, and reducing the tumor ecosystem to a few of the main components that are expected to be involved in the tumor biology may be enough to establish models with superior predictive power over the conventional 2D mono-cultures of tumor cells [18, 19].

Only two studies used 3D culture for investigate chondrosarcoma drug responses have been published [4, 6]. Both of them used chondrogenic three-dimensional pellet model, which consists to culture high density cells in pellets in a chondrogenic medium composed in particular of growth factors, such as TGFβ3 or BMP6. This culture condition allows long term culture and permits cells to differentiate toward chondrogenic phenotype characterized by synthesis of a hyaline matrix. However, this model required the addition of exogenous growth factors which may interfere with chondrosarcoma biology and drug response. That is why we looked for another 3D culture which does not require the addition of growth factors, but which allows chondrogenic differentiation and provide a pathophysiological context that could replicate the chondrosarcoma microenvironment compared to monolayer cultures in 2D system.

Interestingly, chondrocytes (normal cartilaginous cells) encapsulated in alginate present characteristics closer to native cartilage cells than that cultured in 2D [20]. They re-express an extracellular matrix rich in aggrecan and collagen type II, characteristic of hyaline cartilage tissue [21–24], suggesting that this natural biomaterial may be used for 3D culture of chondrosarcomas. Alginate scaffold has advantages as an animal-free product, non-toxic, biodegradable, and easily usable for embedding and next recovering cells, and with significant stability at room temperature [25]. It is a polysaccharide hydrogel composed of β-d-mannuronic acid and α-l-guluronic acid obtained from particular brown algae species. Alginate comprises 99% of water, but still retains high plasticity and mechanical strength. Gelling occurs almost instantaneously by cross-linking with divalent ions, like Ca^2+^, allowing cell entrapment under physiological conditions [26]. Another advantage of alginate hydrogel is the possibility to recover cells from scaffold with a non-enzymatic solution that dissolves alginate within few minutes, but leaves the cells intact for further processing and/or analysis.

Since we have previously shown the benefit to use 3D alginate culture to favor chondrocyte differentiation and to study their biology, we hypothesized that a similar model could permit to culture chondrosarcomas and preserve their chondrogenic phenotype. In the present study, we evaluated the use of this 3D culture system to study chondrosarcoma biology and predict drug response. We validated as null hypothesis that cisplatin has no cytotoxicity in this 3D model, before testing another putative anti-tumoral drug, namely DZNep, which has been shown to induce apoptosis in chondrosarcomas cultured in monolayer. Cytotoxicity in 3D models was compared to in vivo results.

## Methods

### Cell culture

CH2879 [27], JJ012 [28], and SW1353 (from ATCC) chondrosarcoma cell lines were cultured in Roswell Park Memorial Institute 1640’s medium (RPMI 1640) or Dulbecco’s Modified Eagle Medium (DMEM) (Lonza AG, Verviers, Belgium), respectively, supplemented with 10% (*v*/v) fetal bovine serum (FBS) (Lonza AG), penicillin and streptomycin, and then incubated at 37 °C in a humidified atmosphere containing 5% CO_2_. Cells were passaged twice a week.

For 3D culture, cells were suspended at a density of 2 × 10^6^ cells/mL in sodium alginate. Beads were formed as previously described [20–22] by dispensing drops of the suspension from a 22-gauge needle in sterile CaCl_2_ 100 mM. Thereafter, the beads were washed with NaCl 0.15 M and incubated in DMEM or RPMI according to chondrosarcoma cell lines. Photographies were taken using AxioCam MRc5 camera (Zeiss) under VisiScope series 400 microscope (VWR). For some experiments, beads were dissolved using a dissociation solution (55 mM sodium citrate, 150 mM NaCl) before cell harvesting by centrifugation.

### Viability and proliferation assay

Viability and proliferation of chondrosarcomas were estimated for 8 weeks after cell-embedding in alginate beads by measurement of ATP, using the Cell Titer-Glo luminescent cell viability assay kit (Promega, Charbonnieres les bains, France). Cells were lysed directly inside beads according to the manufacturer’s instructions. Luminescence was measured using Victor 3 1420 Multilabel Counter (Perkin Elmer, Villebon-sur-Yvette, France). All experiments were repeated 4 times.

Alternatively, cells were counted after bead dissociation. Ten alginate beads were dissolved, and cells were gently harvested. Then, viable cells were counted using Countess II (Life Technologies) after trypan blue exclusion. Three independent experiments were performed.

### Apoptosis assay

After dissolution of alginate, cells were stained with phycoerythrin (PE)-conjugated antibody directed against APO2.7 (clone 2.7 7A6) according to the manufacturer’s condition (Beckman Coulter, Villepinte, France) as previously reported [29]. Cell fluorescence was measured using Gallios flow cytometer (Beckman Coulter, Villepinte, France) on the technical platform of SFR 146 (Structure Federative de Recherche 146, Caen, France). A minimum of 10,000 events were analyzed in each sample using Kaluza 1.5a software. Three independent experiments were performed.

### RNA isolation and real-time reverse transcription-polymerase chain reaction (RT-PCR)

RNA were extracted using Trizol (Invitrogen, Cergy-Pontoise, France). For monolayer cultures, Trizol was directly added to cell layer in the culture dish. For 3D culture, ten alginate beads were dissolved in 55 mM sodium citrate, 150 mM NaCl and gently centrifuged, before adding Trizol on the cell pellet. Next, extraction was performed according to the manufacturer’s conditions (Invitrogen, Cergy-Pontoise, France). Thereafter, RNA (1 μg) was treated with DNAse-I (Invitrogen, Cergy-Pontoise, France), and reverse transcribed into cDNA in the presence of oligodT and Moloney murine leukemia virus reverse transcriptase (MMLV-RT). The reaction was carried out at 37 °C for 1 h followed by a further 10-min step at 95 °C. Amplification of the generated cDNA was performed by real-time PCR in Step One Plus Real Time PCR apparatus (Applied Biosystems) with appropriate primers. The relative mRNA level was calculated with the 2^–ΔΔCT^ method.

### Cell invasion assay

To evaluate invasion ability of chondrosarcomas, cells were embedded in alginate beads. After 1, 2 or 3 weeks of 3D cultures, beads were transferred in a new culture dishes (15 or 20 beads/well), then incubated for additional 4 days. At the end of that culture period, cells adhering to the bottom of the culture dishes were counted. Invasion ability was evaluated as the number of adherent cells divided by number of beads present in the culture dish. All experiments were repeated three times.

### Drug treatments and cell viability

Cisplatin [*cis*-diammineplatinum(II) dichloride] was purchased from Sigma Aldrich (St Quentin Fallavier, France) and dissolved in DMSO. DZNep-HCl was purchased from Tocris (Lille, France) and dissolved in PBS.

Based on previous works, cells were treated for 3 days with cisplatin (10 μM), or for 14 days with DZNep (1 μM). The medium was changed twice during the treatment of DZNep (day 5 and 10). This condition has been defined as dose and time-treatment sufficient to induce apoptosis in chondrosarcomas. Experiments were performed in 96-well plates (1 bead per well).

After treatments, cell viability was estimated using the Cell Titer-Glo luminescent cell viability assay kit. Luminescence was measured using Victor 3 1420 Multilabel Counter (Perkin Elmer, Villebon-sur-Yvette, France).

### Xenograft of chondrosarcomas in nude mice

Animal experimental procedures were performed according to local legislation, and procedures were approved by ethics committee (Comité d’Ethique de Normandie en Matière d’Expérimentation Animale, agreement #03968.01). Mice were provided and kept in the animal facility (Centre Universitaire de Ressources Biologiques, Caen, France) under controlled temperature and light conditions (temperature 23 ± 2 °C, 12 h reversed light-dark cycle). Animals had ad libitum access to food and water. Each animal was humanely handled throughout the experiment in accordance with internationally accepted ethical principles for laboratory animal use and care, and all efforts were made to minimize animal suffering. Euthanasia was performed using CO_2_ inhalation.

*Nude* mice (11 weeks old, males) were injected subcutaneously with 100 μl of matrigel containing 10^6^ JJ012 cells. When the tumors were palpable, mice were treated by peritoneal injection for 25 days. Tumors were measured by a caliper and tumoral volume calculated by the following eq. (L x w^2^) /2 (with L corresponding to length and w to width).

## Results

### Chondrosarcomas embedded in alginate survived for at least two months

**Fig. 1:**
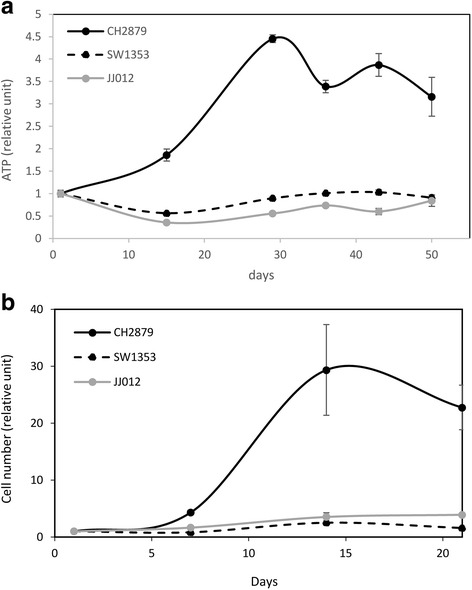
Chondrosarcomas survives in alginate beads. Chondrosarcomas were embedded in alginate beads and viability were evaluated for several weeks. **a** Metabolic activity was evaluated by ATP assay using Cell Titer-Glo luminescent cell viability assay kit (Promega). For each cell line, values were normalized to luminescence values obtained at day 1. Graph shows means ± SEM of 4 independent beads. **b** Viable cells inside beads were also counting. Cell number was normalized to values obtained at day 1. Graph shows means ± SEM of 3 independent experiments

First, we investigated the proliferation of chondrosarcomas embedded in alginate. Three different cell lines (CH2879, JJ012 and SW1353) were embedded at 2 million cells/mL alginate. Then, viability was evaluated by metabolic assay (ATP measurement). All cell lines survived for at least 8 weeks. However, they did not proliferate in beads, except for CH2879 which did it for 4 weeks (Fig. 1a). Counting of viable cells inside beads corroborated that CH2879 cells proliferated faster than the other chondrosarcoma cell lines (Fig. 1b).

### Chondrosarcomas embedded in alginate produced a hyaline matrix

**Fig. 2:**
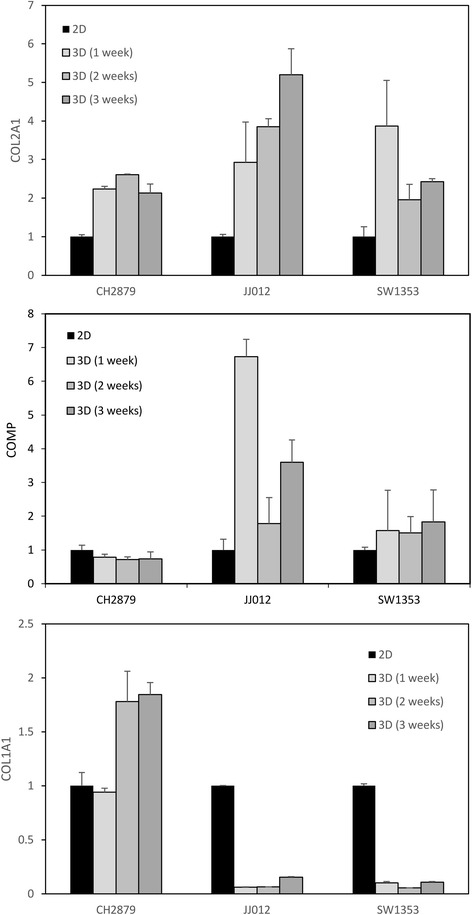
3D culture in alginate favors expression of collagen type II by chondrosarcomas. Chondrosarcomas were cultured in monolayer, or embedded in alginate for 1, 2 or 3 weeks. Then, alginate was dissolved and RNA extracted. Collagen type II and type I, and COMP mRNA levels were assayed by real-time RT-PCR after normalization to RPL13 signal. Values are the mean ± SEM of triplicate experiments

Macroscopically, we observed a clouding/opacification of beads cellularized with JJ012 and SW1353 cells. This white appearance of beads suggests that these chondrosarcomas produced a hyaline-like matrix. To validate this hypothesis, we investigated the expression of the major marker of hyaline cartilage matrix, namely type II collagen. In agreement with macroscopic observation, chondrosarcomas embedded in alginate expressed higher level of type II collagen (Fig. 2a). In addition, JJ012 and SW1353 also increased the expression of cartilage oligomeric matrix protein (COMP, also known as thrombospondin-5), a hyaline ECM gene also known to be more expressed in chondrosarcoma tumors than in tumor derived-cells cultured in monolayer [6]. In contrast, the expression of collagen type I, which is expressed in dedifferentiated chondrocytes and fibrocartilage, was lower in JJ012 and SW1353 beads cultured in 3D compared to 2D. This indicates that 3D culture of CHS in alginate favors the production of a hyaline-like matrix compared to 2D culture, and permits re-expression of genes which are normally present in tumor.

### Alginate culture model allows evaluation of cell invasion or migration ability

**Fig. 3:**
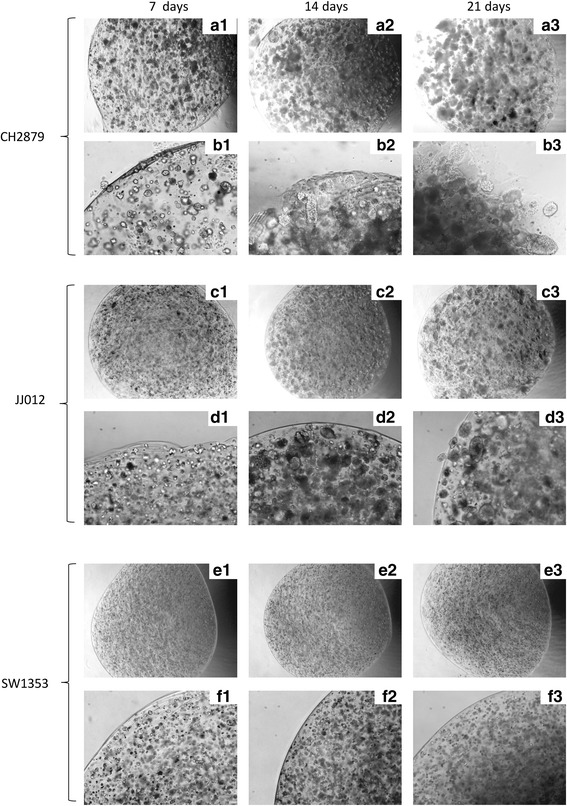
Morphology of beads containing CH2879 differs from other beads. Chondrosarcomas CH2879 (**a** and **b**), JJ012 (**c** and **d**) and SW1353 (**e** and **f**) were cultured in alginate beads for 1, 2 or 3 weeks. Beads were photographed each week. Representative pictures (magnification ×4 (**a**, **c** and **e**) and ×10 (**b**, **d**, and **f**)) are showed for each cell line

**Fig. 4:**
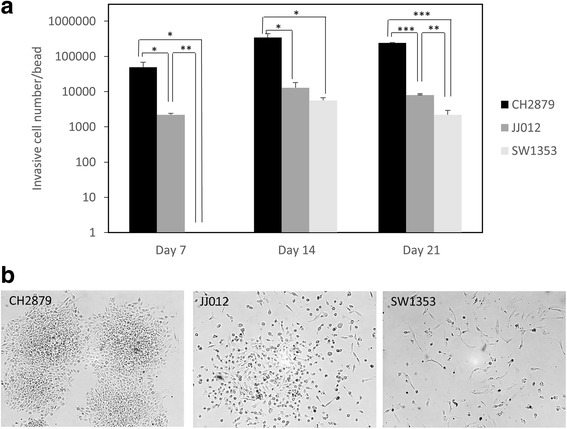
CH2879 cells are more invasive than JJ012 and SW1353. Chondrosarcomas were cultured in alginate beads for 3 weeks. Next, beads were transferred in a new well, and four days later, adherent cell were counted (**a**) and photographed (**b**). Values represent means ± SEM of three independent wells containing each 15–20 beads. *: *p*-value <0.05; **: *p*-value <0.01; ***: *p*-value <0,001

Bead observations revealed that CH2879 cells tended to escape from beads, suggesting invasion or migration abilities (Fig. 3). To investigate this hypothesis, we compared the number of cells outgoing from the beads after 1, 2 and 3 weeks (Fig. 4). This assay revealed a strong heterogeneity of invasion/migration ability according to chondrosarcoma cell lines. Whatever the time of culture, CH2879 cells were much more invasive than the two other cell lines tested. This is consistent with the grade of chondrosarcomas. CH2879 is, indeed, derived from a grade 3 chondrosarcoma (which is known to be very invasive), whereas SW1353 and JJ012 are derived from grade 2 chondrosarcomas (less invasive).

### Chondrosarcomas cultured in 3D were resistant to cisplatin

**Fig. 5:**
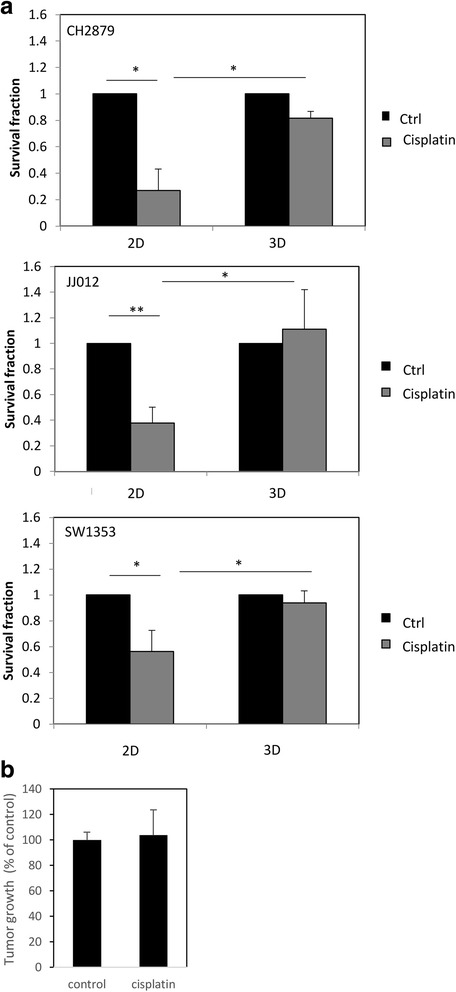
Cisplatin does not reduce the viability of chondrosarcomas embedded in alginate or engrafted in nude mice. **a** Chondrosarcomas cultured in monolayer or embedded in alginate for at least 2 weeks were treated with cisplatin (10 μM) for 3 days. Then, viability was evaluated and compared to untreated cells. Values represent means ± SEM of 3 independent experiments. *: *p*-value <0.05; **: *p*-value <0.01. **b** *Nude* mice were injected with 100 μl of matrigel containing 10^6^ chondrosarcoma cells. When the tumors were palpable, mice were treated for 25 days with cisplatin (i.p., 2 mg/kg, three times per week). Tumors were measured by a caliper and tumoral volume calculated by the following eq. (L x w^2^) /2 (with L corresponding to length and w to width)

Next, we compared the sensitivity of chondrosarcomas to drugs as a function of their culture methods. First, we assayed the toxicity of a widely used chemotherapeutic agent for solid tumors, namely cisplatin. Cisplatin (Pt(NH_3_)_2_Cl_2_, as called cis-diamminedichloroplatinum(II)) is a platinum-based drug, approved as an anticancer agent since 1978, which causes apoptosis by DNA cross-linking. Whereas it is efficient against many cancers, it is not able to treat chondrosarcomas. This resistance has been attributed to the production of an abundant matrix by chondrosarcomas that cannot be mimicked in monolayer cultures [4, 6]. So, we initially tested the null hypothesis that cisplatin has no toxicity in chondrosarcomas cultured in 3D [6]. We then compared cell survival of chondrosarcomas cultured in monolayer or embedded in alginate (Fig. 5a). As expected, cell viability was strongly reduced by cisplatin when chondrosarcomas were cultured in 2D, but not in 3D. Accordingly, cisplatin-induced apoptosis was strongly reduced when cells were embedded in alginate compared to monolayer cultures (Table 1). This is consistent which the absence of toxicity of cisplatin observed in chondrosarcoma xenografts in *nude* mice (Fig. 5b).

**Table 1:** Comparison of apoptosis induced by cisplatin in chondrosarcomas cultured in 2D and 3D cultures

	Apoptotic cell fraction		*p*-value (cisplatin Vs control)	*p*-value (cisplatin 3D Vs cisplatin 2D)
	Control	Cisplatin		
2D	1.8 ± 0.4%	59.4 ± 9.1%	0.009	
3D	25.8 ± 1.9%	38.8 ± 1.9%	0.02	0.03

Chondrosarcoma cells were cultured in monolayer (2D) or embedded in alginate (3D). Then, cells were treated with 10 μM cisplatin. Three days later, apoptosis was assayed. Values represent means ± SEM of 3 experiments

### DZNep reduced survival of chondrosarcomas, even cultured in 3D

We pursued by testing the effect of 3-deazaneplanocin (DZNep), a new anticancer drug (still in preclinical stage), on chondrosarcoma survival [29, 30]. This adenosine analog is a potent inhibitor of S-adenosylhomocysteine hydrolase, resulting in cellular accumulation of S-adenosylhomocysteine, which in turn causes inhibition of S-adenosyl-L-methionine-dependent methyltransferases, particularly enhancer of zeste homolog 2 (EZH2), leading to apoptosis of tumoral cell by a mechanism not yet totally elucidated [29, 30].

**Fig. 6:**
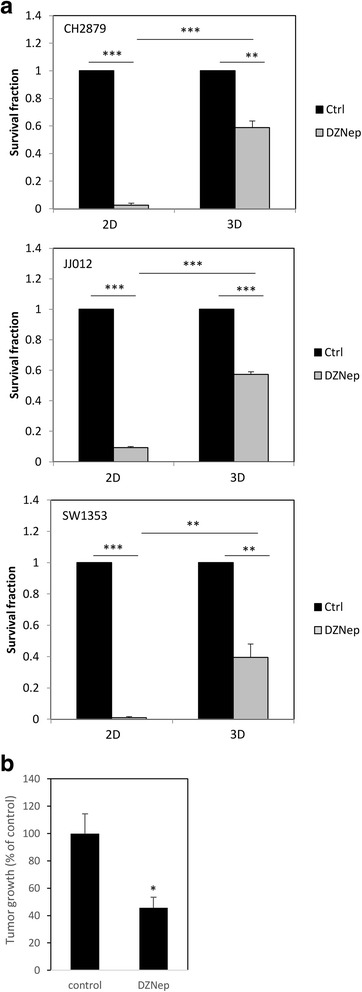
DZNep reduces viability of chondrosarcomas embedded in alginate and engrafted in mice. **a** Chondrosarcomas cultured in monolayer or embedded in alginate for at least 2 weeks were treated with DZNep (1 μM) for 14 days. Then, viability was evaluated and compared to untreated cells. Values represent means ± SEM of 3 independent experiments. **: *p*-value <0.01; ***: *p*-value <0,001. **b** Tumors were implanted as previously (Fig. 4b) and treated with DZNep (i.p., 2 mg/kg, three times per week). Tumors were measured by a caliper and tumoral volume is showed. *: *p*-value <0.05

Interestingly, in contrast to cisplatin treatment, DZNep reduced cell survival and induced apoptosis in chondrosarcomas cultured in 3D (Fig. 6a and Table 2). These results are coherent with in vivo observations showing that DZNep is able to reduce tumoral volume of chondrosarcoma xenograft in nude mice (Fig. 6b).

**Table 2:** Comparison of apoptosis induced by DZNep in chondrosarcomas cultured in 2D and 3D cultures

	Apoptotic cell fraction		*p*-value (DZNep Vs control)	*p*-value (DZNep 3D Vs DZNep 2D)
	Control	DZNep		
2D	0.73 ± 0.3%	29.6 ± 1.4%	0.001	
3D	21.7 ± 1.4%	86.8 ± 1.7%	0.001	0.001

Chondrosarcoma cells were cultured in monolayer (2D) or embedded in alginate (3D). Then, cells were treated with 1 μM DZNep. Fourteen days later, apoptosis was assayed. Values represent means ± SEM of 3 experiments

## Discussion

Cancer researchers typically rely on 2D in-vitro studies and small animal models to investigate the complex mechanisms of tumor invasion and anticancer drug response. However, 2D culture systems often fail to mimic the natural tumor microenvironment, such as cell–cell communication and cell-extracellular matrix interaction, which is essential in tumorigenesis and drug response. Meanwhile, animal models are more accurate representative of tumor environment, but are difficult to use when several conditions are required. That is why there is need to develop relevant preclinical models reproducing natural tumor microenvironment, and easily usable for large scale screening. In particular, chondrosarcomas, as chondrocytes, tend to lose their ability to produce hyaline cartilaginous matrix in monolayer cell cultures. In this paper, we describe a new 3-dimensional culture method using alginate beads, which has advantage to permit hyaline cartilage production by chondrosarcomas without adding any growth factor, and to easily investigate invasion/migration ability and drug response of tumor cells. This model provided a predictive in vitro cell-based assays to study cancer drug efficacy in chondrosarcomas.

Based on our previous works showing the benefit to culture cells in alginate scaffold to favor production of hyaline cartilage matrix [21, 22], we developed a close strategy for in vitro reconstruction of chondrosarcoma microenvironment. We demonstrated here that alginate microencapsulation of chondrosarcoma cells offers numerous advantages. First, it is a robust culture system that allows long term culture, which permits the re-expression of a hyaline-like matrix by chondrosarcomas. Also, this scaffold is easy to use. Cells can be quickly embedded inside alginate, and next used for analyses. Invasion/migration ability as well as drug toxicity can be easily evaluated. Also, if necessary, cells can be recovered by alginate dissociation using a non-enzymatic solution, for additional analysis.

Other scaffolds, such as type I collagen or matrigel, could be used for chondrosarcoma cells. However, they have limitations, including batch-to-batch variation and an incomplete understanding of their impact on cell behavior [31, 32]. In contrast, hydrogels such as alginate present many advantages over bioactive scaffolds due to their inert properties, biocompatible gelation and ease of cell recovery. Thus, analysis of gene expression or cell survival can be done directly from beads or after alginate dissolution. In addition, we developed here a simple strategy permitting to easily evaluate invasion ability of tumor cells, directly by counting cells escaping from alginate beads.

Alginate hydrogels also provide the possibility of conjugation with defined adhesion ligands or delivery of specific biomolecules (growth factors, pro-angiogenic factors, amongst others) [32], and can be used to co-cultured cells. For instance, Brito and collaborators developed alginate embedded culture of epithelial tumor cells aggregated together with human fibroblasts [33]. Such modifications and co-cultures may be made, in the future, to improve this 3D culture system in order to get again closer to natural tumoral microenvironment.

In this report, we show that 3D culture using alginate hydrogel, permits to evaluate invasion/migration ability of chondrosarcomas, and drug toxicity. Thereby, we evaluated toxicity of two anticancer drugs in chondrosarcomas. First, we used cisplatin, which is a common and effective anticancer drug used to in a large variety of tumors (ovaries, testes, lung, solid tumors…), but not efficient to treat chondrosarcomas [34]. Cisplatin is a potent DNA damaging inducer, leading apoptosis in cancer cells [35]. Mechanisms of chondrosarcoma resistance to cisplatin are not yet completely understood, but it is generally admitted that this resistance is linked, at least in part, to the abundant hyaline cartilage matrix rounding chondrosarcoma cells, which strongly reduces drug accessibility to cells [5, 6]. As expected, chondrosarcomas encapsulated in alginate, produce a hyaline-like matrix and are not sensitive to cisplatin, contrary to cells cultured in monolayer (2D).

Next, we tested efficiency of DZNep. This drug has recently been used for its ability to inhibit EZH2 methylase and induce death of tumoral cells in vitro and in vivo [30]. We have previously shown that this pharmacological molecule induces apoptosis of chondrosarcomas cultured in 2D [29]. Before performing in vivo experiments using xenograft mouse model, we wanted to test its efficiency in our more accurate in vitro model with especially hyaline like-matrix production. We observed that DZNep reduced chondrosarcoma survival in this model, suggesting that, contrary to cisplatin, DZNep could be efficient in vivo despite the presence of hyaline matrix. This was confirmed by xenograft experiments in mice showing that, while cisplatin does not modify tumor growth, DZNep is able to reduce it in vivo.

## Conclusion

Three-dimensional in vitro cultures are recognized for recapitulating the physiological microenvironment and exhibiting high concordance with in vivo conditions. Taking the advantages of 3D culture using alginate hydrogel, we have developed an in vitro tumor model for anticancer drug screening. This system has advantages to permit chondrosarcoma cells to produce a hyaline like-matrix, and offers an easy way to assay cell invasion and survival in long term cultures. The developed model system can be transferred across other type of tumors and will provide a new tool for characterization of tumor progression, drug resistance mechanisms and screening new anticancer drugs in vitro.

## Abbreviations

2D: Two-dimensional
3D: Three-dimensional
CHS: Chondrosarcoma
COMP: Cartilage oligomeric matrix protein
DMEM: Dulbecco’s Modified Eagle Medium
DZNep: 3-deazaneplanocin
ECM: Extracellular matrix
EZH2: Enhancer of zeste homolog 2
FBS: Fetal bovine serum
MMLV-RT: Moloney murine leukemia virus reverse transcriptase
PE: Phycoerythrin
RPMI 1640: Roswell Park Memorial Institute 1640’s medium
RT-PCR: Real-time reverse transcription-polymerase chain reaction
